# Scientific Banana Republics: Do They Exist?

**DOI:** 10.5195/cajgh.2016.249

**Published:** 2016-08-03

**Authors:** Faina Linkov, Shalkar Adambekov, Sharon Goughnour, Sharon C. Welburn, Nicolas Padilla-Raygoza, Musa Kana, Eugene Shubnikov, Mustapha M. Mustapha, Aamir Sheikh, Ronald LaPorte

**Affiliations:** 1 Department of Ob/Gyn, University of Pittsburgh School of Medicine, Pittsburgh, PA,USA;; 2 Department of Epidemioloy, Graduate School of Public Health, University of Pittsburgh, Pittsburgh, PA, USA;; 3 Department of Nursing and Obstetrics, Division of Health Sciences and Engineering, University of Guanajuato, Mexico;; 4 EPIUnit, Institute of Public Health, University of Porto, Porto, Portugal;; 5 Department of Community Medicine, Faculty of Medicine, Kaduna State University, Kaduna, Nigeria;; 6 Institute of Internal Medicine, Novosibirsk, Russian Federation;; 7 Center for Innovative Antimicrobial Therapy School of Medicine, University of Pittsburgh, Pittsburgh, PA, USA;; 8 Lincoln Memorial University, Harrogate, TN, USA

The history of the first banana republic begins with the introduction of the banana to the US markets in 1870, when bananas were brought from Jamaica and sold in Boston at a 1,000 percent profit.^1^ Not long after the banana’s introduction, this novel nutritious tropical fruit became much cheaper than the locally grown fruits. The global appetite for bananas lead Jamaica, as well as several other Caribbean countries, to reinvent their economies to those almost solely dependent on the banana production by the end of the 19^th^ century.^2^ The term “banana republic” was introduced by American writer O’Henry to describe the fictional Republic of Anchuria in the book *Cabbages and Kings* (1904),^3^ a collection of short stories inspired by his experiences in Honduras at the turn of the 20^th^ century. Global demand for bananas during that time period led to several international fruit companies controlling the road, rail, and port infrastructure of Honduras. Depending largely on banana exports, economies of countries cultivating the precious crop became fragile, vulnerable, unstable, and much dependent on foreign influence.^4^ Since the times of O’Henry, “banana republic” or “banana state” became a political science term for unstable economies that are largely dependent on exporting a limitedresource product such as bananas.

Though the times of the first banana republics have long passed, the concept has not lost its validity in modern times. Economies of many developing countries around the world are still dependent on exporting a limited resource raw products to wealthier states.^5^ It is not uncommon for the raw product exported by the developing country to be transformed into a commercial commodity in the developed country, and subsequently imported back to the developing country as a new commercial product.^6^

However, the term transcends beyond the fields of economics or political science. As scientists, we are no foreigners to the banana republic concept, with Petsko suggesting that the concept may be relevant to the biological research.^7^ Scientific data produced around the world in forms of databases, laboratory experiments, publications, reports, policies, etc., can be considered as exchange products that can be transported and cultivated from country to country.^8^ Just like fruit producers can be enticed to produce and export one agricultural product that is in high demand; scientists can be tempted to focus on one research topic, for example, Zika, HIV/AIDS, or malaria. The reality is that just like the businessmen, scientists rely on income from local and global funding agencies to “cultivate” their scientific products. Sadly, in many developing countries, relying on local research resources is not always enough to produce quality research and this is where international sponsors and collaborators play a huge supporting role. International sponsors, funding agencies, philanthropists, etc. contribute tremendously to health research and improvement in both developed and developing world.^9^ For example, the Gates foundation funded much needed polio, HIV, and malaria prevention programs on the African continent.^10^ Similarly, the National Institutes of Health (NIH) in the US funds a number of efforts in the area of infectious disease in Africa. While it is wonderful for the developing countries to get this financial aid to promote their research, the potential pitfall is that some of the health needs of these countries (such as chronic disease) may get overshadowed and not receive much needed support.

For example, out of the 100 recently funded NIH awards targeting Africa, close to 60% are associated with infectious diseases, such as HIV, tuberculosis, and malaria.^11^ The remaining grants are aimed maternal and child health, education, and nutrition and not necessarily chronic diseases, which are the key cause of age related morbidity in the majority of African countries.^12^ NIH funds, as well as funds from other agencies, provided for conducting research in the developing world are really important, as they not only support research but also improve laboratory infrastructures (as applicable) and establish training programs for the researchers in the developing world.^11^ Without such support, many important discoveries would not be possible. On the other hand, relying heavily on foreign aid in supporting research may be limiting scientific diversity in the developing world by focusing the research on a relatively small group of specific research topics.

For example, with Zika, NIH supported a national scientific research organization linked to the Brazilian Ministry of Health, to conduct a multi-country study to evaluate the magnitude of health risks that Zika virus infection poses to pregnant women and their developing fetuses and infants.^13^ While Zika is a very important disease to investigate, these particular funding mechanisms potentially encourages study sites in Puerto Rico, Brazil, Colombia, and other areas that are experiencing active local transmission of the virus to depend on NIH to conduct a very specific line of infectious disease research. On one hand, research infrastructure of countries affected by Zika will benefit from these resources. On the other hand, the research priorities of these developing regions are potentially determined not by local policy makers or researchers, but by funding agencies of the developed world.

As a consequence, what we are observing is that the dependence on much needed foreign grants to investigate major public health concerns in the developing world establishes the concept of Scientific Banana Republic. A Scientific Banana Republic is a country that is very limited in the kind of biomedical (or scientific) research it can do, due to mostly focusing on producing and exporting raw research data, predominantly in the various fields of infectious diseases. This concept is related to a well published concept of Scientific Imperialism and Safari Research, where science may be dominated by the scientific interests of developed countries. Table 1 reviews similarities between agricultural and scientific banana states.^14^

Similarly, if we explore scientific productivity in Africa in the form of scientific publications, we see that publication rate follows funding areas very closely. If we explore the top 100 research articles recently produced by the African continent (using Web of Science), we can see that a large portion (35%) of these articles focus on infectious diseases especially HIV/AIDS. Moreover, scientists from these countries rely heavily on international collaborations to publish their results instead of collaborating with local institutions.^15^ As a result, such countries produce “raw” scientific product, which is oftentimes transformed into “final product” (published research) by scientists in developed countries.^16^ Increasing research collaboration between industrialized and developing countries also raised concerns about ethical review process of the US and international ethics review boards. A survey of health researchers from developing and developed countries suggested a desire for focused capacity development in supporting local ethical review of research with effective implementation of culturally appropriate studies in the developing world.^17^

Globalization and its consequences have many implications for health locally and globally. The concept of global health has become extremely popular in the past two decades, as the global thirst for knowledge on old diseases and newly emerging infections is increasing. New global health degrees and certificate programs open each day in US schools and around the world. Global health is becoming increasingly more interesting to public health and medical workers, especially with the Zika virus in Brazil, the continued HIV epidemic along with outbreaks of Ebola in sub-Saharan Africa, and the global threat from malaria. Scientists in countries, oftentimes impoverished, that are affected by these diseases obtain international funds to explore these problems and share this knowledge with the rest of the world. Is this good or bad?

Indeed, HIV and malaria are problematic for many African countries causing many preventable deaths. Despite the fact that infectious diseases are extremely important problems that should be investigated, analysis of top causes of deaths for countries with a high HIV burden such as South Africa and Zimbabwe reveal that chronic diseases such as stroke and cardiovascular disease are found in the top 5 leading causes of death. In addition, trauma associated deaths are very important in the developing countries, leading to the “triple burden of disease” commonly experienced by developing countries undergoing epidemiologic transition.^18^ Thus, exploration of chronic diseases and trauma in the developing world is very important and relevant to improving health locally and globally. The focus on infectious disease in the developing world, without taking into account chronic disease and trauma may not comprehensively address the health challenges of the 21^st^ century. In political science, we learned to recognize the dangers of agricultural banana states. We would like to argue that in the 21^st^ century, it is time for us to recognize the problems potentially associated with scientific banana republics.

There are several possible solutions to the problems identified above. One of the common myths associated with healthcare in developing countries is that that their healthcare systems are not equipped to handle the triple burden of disease. However, numerous examples demonstrate how by using grassroots approaches and relying on local inexpensive public health interventions, public health officials in the developing countries were able to make significant progress by using local resources.^19^,^20^ Thus, mobilizing existing resources to improve local cost effective and culturally appropriate interventions is one of the best solutions to improving “local” health. Local resources can be shared through global networks, such as the Supercourse (www.pitt.edu/~super1) or the Research Methods Library of Alexandria (http://ssc.bibalex.org/helpdesk/introduction.jsf), both of which help investigators with research methods, statistical analysis education, and publications. New journals, such as the Central Asian Journal of Global Health (http://cajgh.pitt.edu/), target publications from scientists in under-served countries by providing mentorship and guidance to authors who may have difficulties in publishing their research findings in English speaking journals. It is important for governments of developing countries to allocate more funds for public health. It is also important to encourage discussion between policy makers in developing and developed countries to ensure that global health priorities fit the local health priorities when it comes to funding from international funding agencies. It may also make sense to identify and actively engage local stakeholders in identifying ways to improve scientific productivity in the developing world. Local stakeholders, such as medical doctors, nurses, university researchers, and community representatives, may represent the voices of people that are not typically represented in the policy making process. We would like to conclude this editorial by pointing out that scientific banana republics may be good for the scientific infrastructure of developing countries, as research may not be possible without foreign support. At the same time, we would like to increase awareness of this concept, as without recognizing such concept it may not be possible to effectively address the triple burden of disease in the developing world.

## Figures and Tables

**Table 1.** Agricultural banana state vs. scientific banana state

**Characteristic**	**Agricultural banana republic**	**Scientific banana republic**
Single resource product Raw material export	Banana, orange, and raw materials exported at low prices	Raw data oftentimes sent to be analyzed at Western institutions
Unstable government Stratification of social classes	Oligarchy Large income gaps between people of different classes	Excessive power in the hands of scientific leaders (deans, chairs, etc) Difficulties in climbing academic ladder for junior scientists
Foreign entities supporting infrastructure	Foreign fruit companies supporting railroads and other infrastructure in traditional banana states to support agricultural infrastructure	Foreign funding agencies supporting research, laboratory and other infrastructure for institutions and groups focusing on one or several types of research questions, mostly related to infectious disease
Economy dominated by foreign interest	Economy dominated by international fruit companies	Research topics “suggested” by international funding agencies in the form of requests for applications on specific topics
